# Differential Effects of E2 on MAPK Activity in the Brain and Heart of Aged Female Rats

**DOI:** 10.1371/journal.pone.0160276

**Published:** 2016-08-03

**Authors:** Elena Pinceti, Cody L. Shults, Yathindar S. Rao, Toni R. Pak

**Affiliations:** Department of Cell and Molecular Physiology, Health Science Division, Loyola University Chicago, Maywood, Illinois, United States of America; Universidade de São Paulo, BRAZIL

## Abstract

Aging and the coincident loss of circulating estrogens at menopause lead to increased risks for neurological and cardiovascular pathologies. Clinical studies show that estrogen therapy (ET) can be beneficial in mitigating these negative effects, in both the brain and heart, when it is initiated shortly after the perimenopausal transition. However, this same therapy is detrimental when initiated >10 years postmenopause. Importantly, the molecular mechanisms underlying this age-related switch in ET efficacy are unknown. Estrogen receptors (ERs) mediate the neuroprotective and cardioprotective functions of estrogens by modulating gene transcription or, non-genomically, by activating second messenger signaling pathways, such as mitogen activated protein kinases (MAPK). These kinases are critical regulators of cell signaling pathways and have widespread downstream effects. Our hypothesis is that age and estrogen deprivation following menopause alters the expression and activation of the MAPK family members p38 and ERK in the brain and heart. To test this hypothesis, we used a surgically induced model of menopause in 18 month old rats through bilateral ovariectomy (OVX) followed by an acute dose of 17β-estradiol (E2) administered at varying time points post-OVX (1 week, 4 weeks, 8 weeks, or 12 weeks). Age and E2 treatment differentially regulated kinase activity in both the brain and heart, and the effects were also brain region specific. MAPK signaling plays an integral role in aging, and the aberrant regulation of those signaling pathways might be involved in age-related disorders. Clinical studies show benefits of ET during early menopause but detrimental effects later, which might be reflective of changes in kinase expression and activation status.

## Introduction

Clinical and basic science studies have shown that estrogens are neuroprotective and cardioprotective. Treatment with estrogens (ET) can reduce the incidence of Alzheimer disease, improve survival following ischemic stroke, improve learning and memory, and reduce anxiety and mood disorders [1–7]. Moreover, women are less likely to develop heart disease than men, but this advantage is lost after menopause presumably due to a significant reduction in circulating estrogens [8–10]. Clinical correlates have indicated that the decline of circulating estrogens coincident with menopause leads to an increased risk of coronary heart disease, atherosclerosis, hypertension, stroke, neurodegenerative disease, cognitive decline, and mood disorders. However, the first large scale clinical trial designed to assess the health benefits of ET in postmenopausal women (The Women’s Health Initiative (WHI)) was prematurely suspended because of an unexpected increased risk of stroke and coronary heart disease among the participants [11]. Subsequent analysis of the clinical data revealed that the age of the participants significantly affected the outcome. Women who began ET at the time of menopause or within 10 years, had significant reductions in heart disease and overall mortality [12]. This observation was coined the “Timing Hypothesis”, and postulated that ET administration is beneficial for early postmenopausal women, but detrimental in late postmenopausal women. Importantly, the underlying molecular mechanisms for these observations remain undetermined.

The actions of estrogens are mediated primarily by estrogen receptor (ER) α and ERβ. Both receptors can mediate the neuroprotective and cardioprotective effects of estrogens [3, 6, 13–17]. Estrogens bind ERs to regulate gene transcription through classical genomic pathways, or by modulating cell signaling pathways such as the MAPKs (mitogen activated protein kinases) ERK (extracellular signal-regulated kinase) and p38 [18–25]. Moreover, aging alone modulates similar cell signaling pathways independent of estrogens [21, 24, 26–31]. One possibility for these age-related changes is that MAPKs are sensitive to proinflammatory and oxidative stimuli, which are increased with age [32]. These converging data suggest that MAPK signaling could be a molecular mechanism underlying the discrepant effects of ET in postmenopausal women.

MAPK family members are activated by phosphorylation at their threonine-x-tyrosine phosphorylation site motif. MAPKs activation is the last of a three step activation cascade from MAP3K to MAP2K to MAPK. Once phosphorylated (i.e. activated) they in turn phosphorylate substrate proteins at serine or threonine residues in specific recognition sequences [33]. The knowledge of the activators, substrates and functions of MAPKs are ever expanding, however when they were first discovered they were deemed stress-activated protein kinases (SAPKs) because of their responsiveness to toxins, physical stresses and inflammatory cytokines [34, 35]. Generally, ERK1/2 are preferentially activated in response to growth factors and extracellular stimuli, while p38 is more responsive to stresses such as osmotic shock, ionizing radiation and cytokines [36]. MAPKs can be present in several cell compartments, such as the nucleus, the cytoplasm and close to the cell membrane, thereby allowing them to integrate signals to coordinate a variety of physiological responses including mitosis, apoptosis, survival, cellular differentiation, and gene expression.

Activated MAPKs target a wide pool of proteins due to sequence variation in the conserved phosphorylation motif and in the availability of docking sites on their protein targets. The strongest requirement is the presence of a proline directly at the C- Terminus of the target protein phosphorylation site and this feature is a shared requirement for both p38 and ERK kinases. Another characteristic feature differentiating the downstream effects of MAPKs is the presence of docking sites in their substrates; the best characterized being D-sites and F-sites. ERK and p38 phosphorylate different target proteins, leading to differential downstream effects because of their heterogeneous recognition motif and respective preference for D- and F- sites in the targets [37]. Interestingly 30% of human proteins have at least one MAPK phosphorylation site, yet few have the required docking sites to facilitate phosphorylation by MAPK family members.

The overall goal of this study was to quantify the total expression and activation of MAPKs (ERK and p38) in the brain and heart of aged female rats subjected to a paradigm designed to model the tenets of the Timing Hypothesis. We hypothesized that the combination of age and low circulating estrogens alters the expression and activation of p38 and ERK kinases in the brain and heart. To test this hypothesis, we used a rat model of surgically-induced menopause and quantified changes in kinase activity following varying lengths of E2 deprivation. Our data revealed age- and E2-dependent effects on kinase activity suggesting a potential mechanism explaining the variable effects of E2 following menopause.

## Methods

### Ethics Statement

Animal procedures were designed to minimize pain and suffering. Euthanasia was performed using inhaled vaporized isoflurane followed by rapid decapitation using a rat guillotine, in accordance with the AVMA Guidelines for the Euthanasia of Animals: 2013 Edition. All animal protocols were approved by the Institutional Animal Care and Use Committee (IACUC) at Loyola University Chicago, permit number 2009018.

### Animals

Female Fischer 344 rats were obtained from the National Institute of Aging (NIA) colony (Taconic) at 18 months (N = 80) of age. The animals were allowed to acclimate to the housing facility for 7 days after arrival. Animals were housed two per cage and were allowed free access to standard rat chow and tap water.

**Fig 1:**
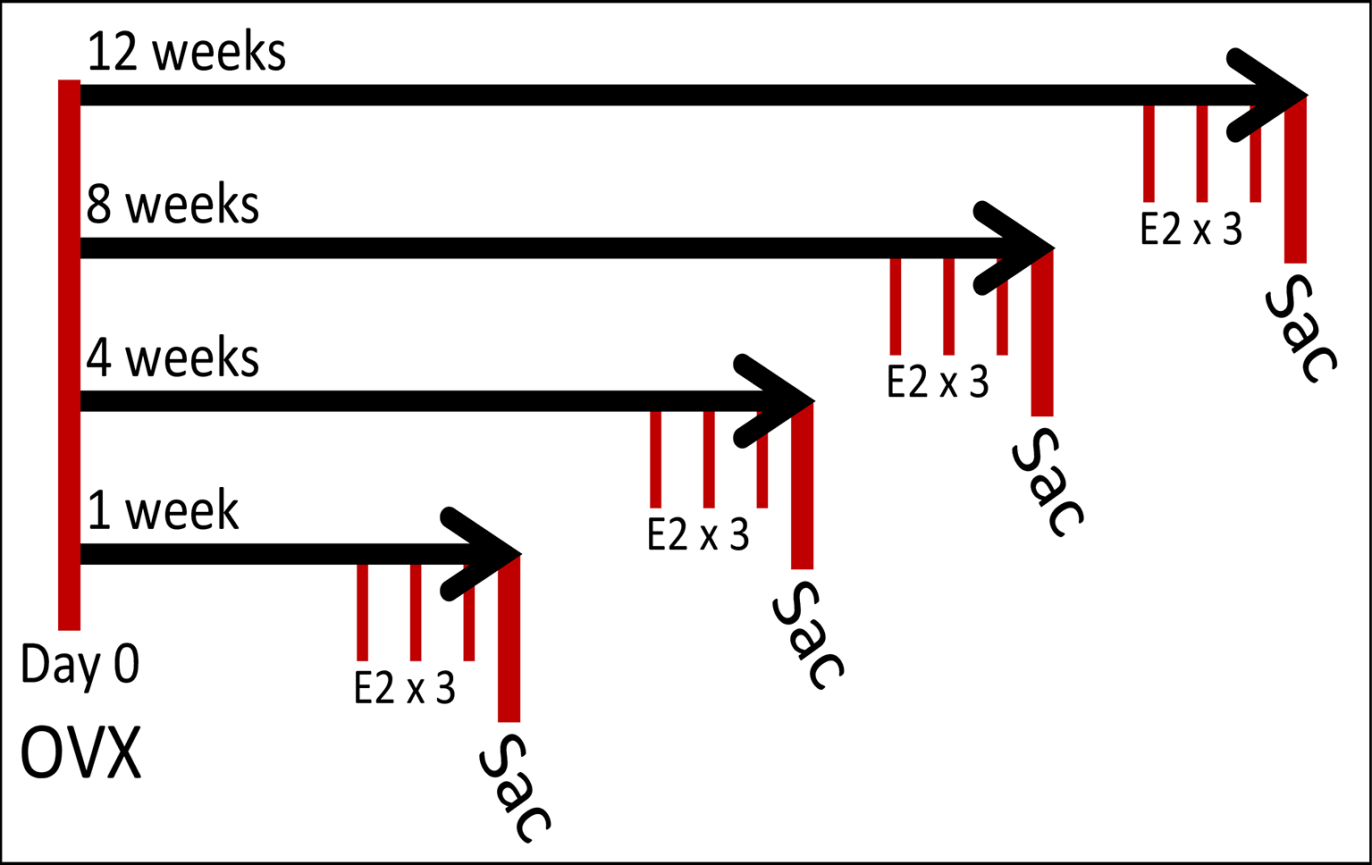
Diagram of the animal paradigm. Female Fisher 344 rats were ovariectomized at day 0 (= 18 months old) and subjected to increasingly longer periods of hormone deprivation (1, 4, 8, and 12 weeks). Following the assigned length of deprivation, animals were treated with either vehicle (safflower oil) or 17β-estradiol (E2; 2.5 μg/kg) by subcutaneous injection once daily for 3 consecutive days (n = 10/treatment group/deprivation time). Animals were euthanized 24 hours following the last treatment.

One week after arrival, animals were deeply anesthetized with vaporized isoflurane and bilaterally ovariectomized (OVX). Briefly, the ovary and distal end of the uterine horn were pulled from the body cavity through a 1 cm incision made through the skin and body wall. The uterine horn was clamped with a hemostat and ligated proximal to the clamp. The entire ovary and distal uterine horn were then removed. Animals were singly housed and provided with acetaminophen analgesic (122.7 mg/kg) in their water for 3 days postoperative. During this time, animals were weighed once/day and their water intake was measured. Following 3 days of analgesia, the animals were pair-housed with their previous cage mate for the duration of the experiment. Following OVX animals recovered for 1, 4, 8, or 12 weeks (N = 20/age group, Fig 1). After the designated recovery time the animals were given a subcutaneous injection of either safflower oil (vehicle) (N = 10/age group) or 2.5 μg/kg 17β-estradiol (E_2_, N = 10/age group) dissolved in safflower oil once/day for 3 days. This treatment paradigm approximately resembles the period of the estrous cycle with higher levels of circulating estrogens. Similar acute treatment paradigms (1x/day for 1 to 4 days) resulted in physiological and behavioral improvements in ovariectomized rats [38, 39] [40] [41–43] [44, 45]. Animals were euthanized 24 hours after the last injection, trunk blood was collected, brain and heart rapidly removed and flash frozen. Trunk blood was centrifuged at 4500 rpm for 8 minutes at 4°C. The plasma samples first underwent a liquid-liquid extraction using diethyl ether to eliminate interfering compounds in the plasma as previously described [46, 47]. After diethyl ether extraction, samples were tested using a high-sensitivity ELISA kit (AD 901 174; Enzo Life Sciences) to determine the concentration of circulating E2 (n = 6/age per treatment) according to manufacturer's specifications. A separate group of 18-month old Fisher 344 rats were left ovarian intact (N = 6) and had low circulating E2 levels (35.0 ± 7.1 pg/mL; n = 6) [47] which was consistent with diestrous-like vaginal cytology, as assessed daily for 2 weeks before euthanasia. Circulating E2 levels remained low in 18-month old animals treated with vehicle 1 week after OVX (23.2 ± 2.7 pg/mL; n = 6) [47]. E2 treatment increased circulating E2 levels in OVX animals (56.5 ± 6.3 pg/mL; n = 6), which is within physiological range of women who receive HT during postmenopause (17–75 pg/mL) [47] [48] [49]. Treatment with E2 increased circulating levels consistently within this range throughout the deprivation paradigm [47].

### Tissue Collection

The hypothalamus, dorsal and ventral hippocampus were microdissected using a 0.75 mm Palkovit’s brain punch tool (Stoelting, Inc., Wood Dale, IL) according to *“The Rat Brain in Stereotaxic coordinates”* (Paxinos et al.1998). The hypothalamus (−0.8 to −3.8 mm relative to bregma), dorsal hippocampus (−2.30 to −4.16 mm relative to bregma), and ventral hippocampus (4.30 to 6.04 mm relative to bregma) were all microdissected for RNA and protein isolation. The left ventricle was also rapidly removed from heart that had been flash frozen and homogenized using silica beads and a Mini Beadbeater-8 (Biospec Products, Bartlesville OK).

### RNA Isolation

Trizol reagent (Invitrogen, Carlsbad CA) was used to isolate total RNA from the hypothalamus, ventral hippocampus, dorsal hippocampus, and left ventricle of the heart. All RNA samples were quantified using Nanodrop spectrophotometry and analyzed for quality by visualization of the RNA on 1.5% agarose gel.

### Quantitative Reverse Transcription PCR (RT-qPCR)

Following RNA isolation, 1.0 μg total RNA was reverse transcribed using the SuperMix VILO cDNA synthesis kit for RT-qPCR (Invitrogen, Carlsbad, CA). Roche FastStart SYBR Green Master Mix was added to intron-spanning ERK and p38 primers: ERK forward: 5’CTCGGATTCCGCCATGAGAA3’, reverse: 5’GGTCGCAGGTGGTGTTGATA3’; p38 forward: 5’CAGGAAACGGGACGAACAGA3’, reverse: 5’CCACAGAACTGCATGTCCCT3’. Then, 2 μL cDNA templates were added to duplicate reactions performed in 96 well plates. The following program was used for RT-qPCR: 1) 95°C for 10 minutes, 2) 95°C for 30 seconds, 3) 59°C for 30 seconds, 4) 72°C for 30 seconds, and melting curve analysis. All samples were normalized to the hypoxanthine guanine phosphoribosyl transferase 1 (HPRT) housekeeping gene (primers: forward: 5’AGCAGTACAGCCCCAAAATGG3’, reverse: 5’TGCGCTCATCTTAGGCTTTGT3’), as it is not altered by E2 treatment [47, 50]. Quantification of the target gene expression was achieved using the ∆∆CT method [51].

### Protein Isolation

Total protein was extracted from the hypothalamus, dorsal hippocampus and ventral hippocampus using T-Per reagent (ThermoFisher Scientific, Waltham MA) supplemented with Pierce Protease and Phosphatase Inhibitor Tablet, EDTA Free (ThermoFisher Scientific, Waltham MA). Similarly, total protein was extracted from the left ventricle of the heart using RIPA buffer supplemented with Pierce Protease and Phosphatase Inhibitor Tablet, EDTA Free (ThermoFisher Scientific, Waltham MA). Protein concentrations were measured using the Pierce BCA Protein Assay kit according to manufacturer’s directions (ThermoFisher Scientific, Waltham MA).

### Western Blot

10 μg of isolated protein was electrophoresed on a 10% acrylamide gel. The gel was then transferred on a PVDF membrane (Promega, Madison WI), blocked for 1 hour with 5% Bovine Serum Albumin (BSA, ThermoFisher Scientific, Waltham MA) in Tris Buffered Saline with 0.1% Tween (TBST), then incubated with primary antibodies in 5% BSA TBST overnight. Primary antibodies: ERK1/2 (1:500 dilution, sc-94, Santa Cruz, Dallas TX), phosphoERK1/2 (1:500, 9101s, Cell Signaling, Danvers MA), p38 (1:500, 8690s, Cell Signaling, Danvers MA), phosphop38 (1:500, 4511s, Cell Signaling, Danvers MA). Blots were washed twice with TBST for 10 minutes prior to application of a secondary antibody (1:5000, 7074s, Cell Signaling, Danvers MA) in 5% BSA TBST for 2 hours. Blots were then washed twice with TBST for 10 minutes and imaged using a Biorad Chemidoc XRS+ imager following application of the Pierce Enhanced Chemiluminescence (ECL) Western Blot substrate (ThermoFisher Scientific, Waltham MA). PonceauS staining (MP Biomedicals, Santa Ana CA) was used to detect total protein. Quantification of bands was achieved by measuring the intensity of the bands following normalization to total protein with ImageLab software. Biological replicates were 6–8 per treatment/time point and samples were repeated in 2–3 technical replicates.

### Statistics

Two-factor ANOVA with Tukey post hoc pairwise comparisons was performed to determine statistical significance and interaction between the groups. When no interaction was found, one-factor ANOVA and t-test were performed. P-value of less than 0.05 was considered significant.

## Results

### Length of E2 deprivation and subsequent E2 treatment alters ERK and p38 kinase activity in the brain and heart of aged female rats

We first tested whether the effects of E2 treatment on ERK and p38 kinase activity would depend on the length of time that the aged animals were deprived of circulating E2 (i.e. time post-OVX). We measured the ratio of phosphorylated:total ERK and p38 protein as a measure of kinase activity in 3 distinct brain regions (hypothalamus, dorsal hippocampus, ventral hippocampus) and in the left ventricle of the heart. The hypothalamus regulates homeostasis, thermoregulation and stress response, which are often dysregulated in post-menopausal women [52]. The hippocampus is functionally divided in two regions, dorsal and ventral [53] and both regions express ERs. The dorsal hippocampus (DH) mediates cognitive functions, while the ventral hippocampus (VH) mediates processes associated with emotional memory and stress [53]. In addition, we focused on the heart because the likeliness of developing heart disease increases in women post-menopause, and sex differences in cardiovascular disease are well characterized. The left ventricle (LV) is the main pumping chamber of the heart and it is most commonly subjected to diseases that accompany aging, such as hypertrophy. Estrogen receptors are highly expressed in the LV and the cardioprotective effects of E2 treatment are evident in the LV [17, 54, 55].

#### Brain–Hypothalamus

**Fig 2:**
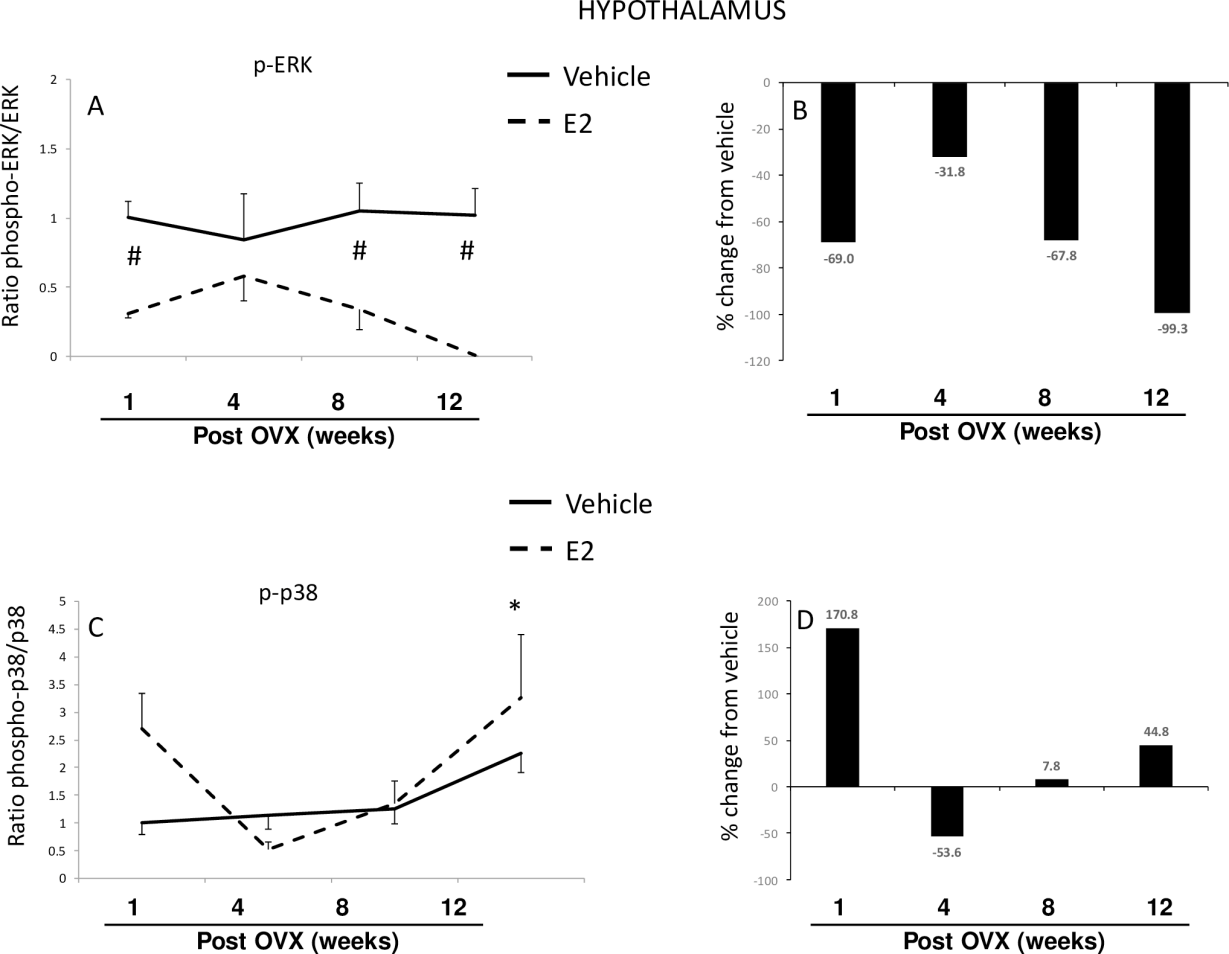
Effects of age and E2 treatment on ERK and p38 activation in the hypothalamus. Calculated ratio of phospho:total-ERK or p38(A, C), and percent change from vehicle following E2 treatment (B, D). Data are expressed as mean fold change ± SEM compared to vehicle-treated animals at one week post-OVX (A, C). An * indicates statistically significant difference from 1-week time point; # indicates significant difference within the same time point.

A two-factor ANOVA analysis showed a significant interaction between the length of E2 deprivation and subsequent E2 treatment in the hypothalamus (all F-values reported in Table 1). The ratio of phosphorylated ERK to total ERK protein was significantly lower at all time points in E2-treated animals (Fig 2A), representing a large magnitude shift from baseline compared to vehicle-treated controls (Fig 2B). This effect was primarily due to changes in the levels of phosphorylated ERK, the active form of the protein, and not total ERK. Specifically, our data showed that E2 treatment significantly decreased the amount of phosphorylated ERK to levels that were barely above detection at the 8- and 12-week post-OVX time points. Conversely, total ERK remained stable at all time points except for an increase observed in E2-treated animals at 12-weeks post-OVX (S1A and S1B Fig).

**Table 1:** Statistical analysis. F and p-values from two-factor ANOVA in hypothalamus, dorsal hippocampus, ventral hippocampus, and heart left ventricle. Light gray shading indicates analysis of ERK and white background indicates analysis of p38.

	Interaction: Time x Treatment	Main Effect: Treatment	Main Effect: Time
**Hypothalamus**			
ERK mRNA	No	No	Yes: F(3, 37) = 3.320, p<0.001
ERK protein	No	No	No
phosphoERK	No	Yes: F (1,39) = 18.226 p<0.001	No
ERK Ratio (total:phospho)	No	Yes F (1,44) = 15.670 p<0.001	No
p38 mRNA	No	No	Yes: F(3, 35) = 6.474, p = 0.001
p38 protein	No	No	Yes: F (3,36) = 11.346, p<0.001
phosphop38	No	No	No
p38 ratio (total:phospho)	Yes: F (3,33) = 3.558, p = 0.025	No	Yes: F (3,36) = 3.821, p = 0.018
**Dorsal Hippocampus**			
ERK mRNA	No	No	Yes: F (3,32) = 4.105, p = 0.014
ERK protein	No	No	No
phosphoERK	Yes: F (3,39) = 4.012, p = 0.014	No	No
ERK Ratio (total:phospho)	No	No	No
p38 mRNA	No	No	Yes: F(3, 33) = 4.214, p = 0.013
p38 protein	No	No	No
phosphop38	No	No	Yes: F(3, 37) = 6.586, p = 0.001
p38 ratio (total:phospho)	No	No	Yes: F(3, 37) = 5.653, p = 0.003
**Ventral Hippocampus**			
ERK mRNA	No	No	No
ERK protein	Yes: F (3, 31) = 2.915, p = 0.050	Yes: F (1, 31) = 6.294, p = 0.018	Yes: F (3, 31) = 12.942, p<0.001
phosphoERK	Yes: F (3, 34) = 3.205, p = 0.035	Yes: F (1, 34) = 4.806, p = 0.035	Yes: F (3, 34) = 3.955, p = 0.016
ERK Ratio (total:phospho)	Yes: F (3, 35) = 3.177, p = 0.037	No	No
p38 mRNA	Yes: F (3, 37) = 6.621, p = 0.003	Yes: F (1, 37) = 7.783, p = 0.008	Yes: F (3, 37) = 6.493, p = 0.001
p38 protein	Yes: F (3, 33) = 4.624, p = 0.008	No	Yes: F (3, 33) = 3.583 p = 0.024
phosphop38	No	No	Yes: F (3, 34) = 4.822, p = 0.007
p38 ratio (total:phospho)	Yes: F (3, 32) = 3.727, p = 0.021	No	Yes: F (3, 32) = 6.713, p = 0.001
**Heart**			
ERK mRNA	Yes: F (3, 35) = 3.385, p = 0.029	No	Yes: F (3, 35) = 8.502, p<0.001
ERK protein	Yes: F (3, 58) = 4.430, p = 0.007	No	Yes: F (3, 58) = 6.806, p = 0.001
phosphoERK	Yes: F (3, 59) = 5.227, p = 0.003	Yes: F (1, 59) = 4.893, p = 0.031	Yes: F (3, 59) = 20.089, p<0.001
ERK Ratio (total:phospho)	No	No	Yes: F (3, 60) = 14.834, p<0.001
p38 mRNA	No	No	Yes: F(3, 35) = 2.385, p<0.001
p38 protein	No	No	No
phosphop38	Yes: F (3, 44) = 3.502, p = 0.023	No	No
p38 ratio (total:phospho)	No	No	Yes: F(3, 51) = 5.299, p = 0.003

By stark contrast to ERK activity in the hypothalamus, the ratio of phospho:total p38 was not significantly altered by E2 treatment at any time point (Fig 2C). Notably however, there were changes in the amount of phosphorylated p38 observed at specific time points. For instance, E2 treatment increased phosphorylated p38 by 170% at 1-week post-OVX compared to vehicle treated animals at that same time point (Fig 2D). Similarly, phosphorylated p38 increased 3-fold in both vehicle and E2-treated groups at the 12-week time point, whereas total p38 protein did not change (S1C and S1D Fig)

#### Brain—Dorsal hippocampus

**Fig 3:**
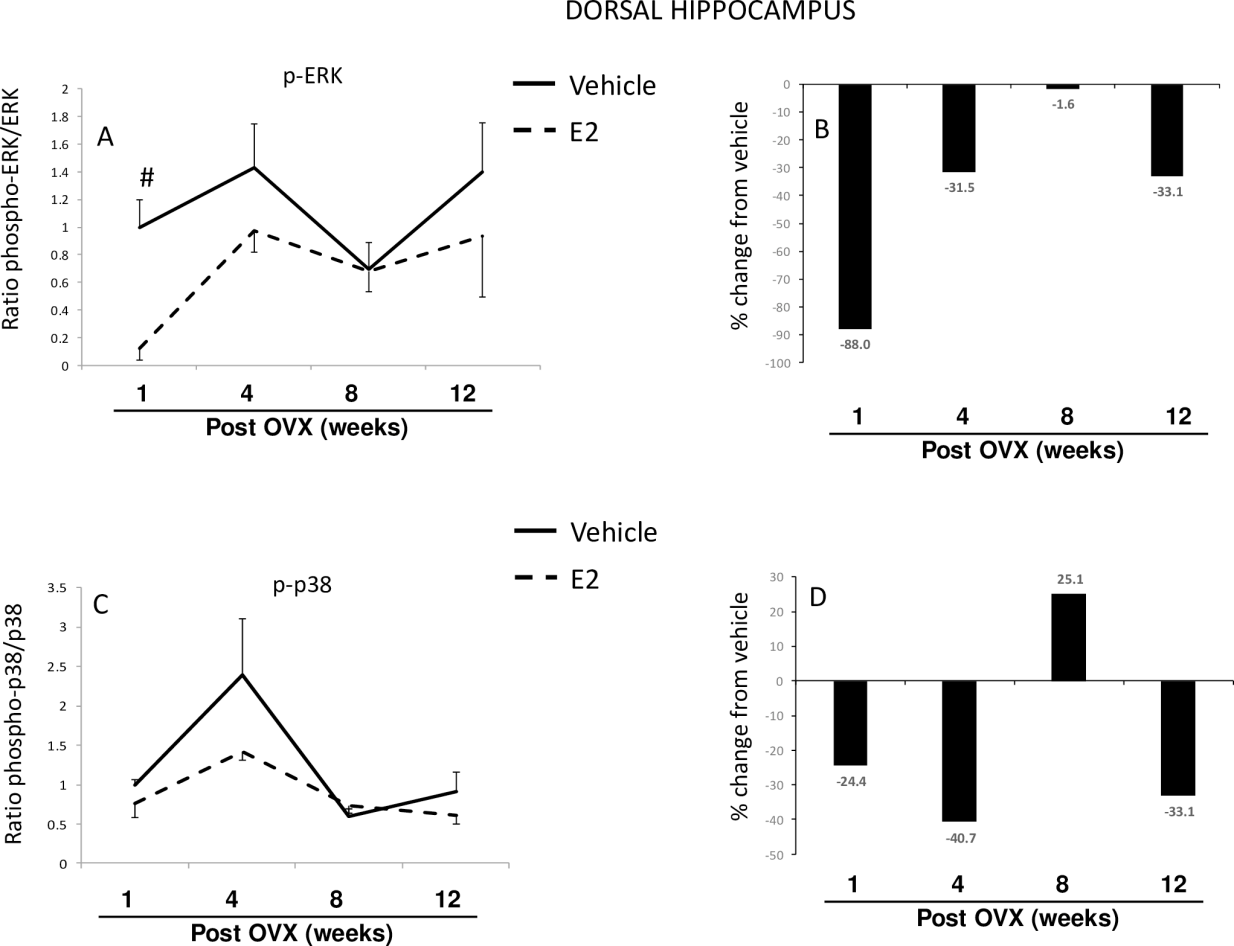
Effects of age and E2 treatment on ERK and p38 activation in the dorsal hippocampus. Calculated ratio of phospho:total-ERK or p38 (A, C), and percent change from vehicle following E2 treatment (B, D). Data are expressed as mean fold change ± SEM compared to vehicle-treated animals at one week post-OVX (A, C). An * indicates statistically significant difference from 1-week time point; # indicates significant difference within the same time point.

We then measured ERK and p38 protein and phosphoprotein in the dorsal hippocampus (Fig 3). Similar to the results observed with ERK in the hypothalamus, a two-factor ANOVA revealed a significant interaction between length of E2 deprivation (i.e. time post-OVX) and subsequent E2 treatment (Table 1). The ratio of phospho:total ERK was significantly lower than vehicle-treated animals at 1-week post-OVX (Fig 3A) and the overall levels of phospho-ERK were consistently lower in E2-treated animals across the treatment paradigm (Fig 3B). E2 treatment significantly decreased phosphorylated ERK at the 1-week time point, yet there were no changes in total ERK protein in either group (S2A and S2B Fig).

Next, we measured p38 activation in the dorsal hippocampus. A two-factor ANOVA analysis revealed a significant main effect of time, but no significant interaction suggesting that the two variables were not dependent on each other (Table 1). The ratio of phospho:total p38 was not significantly different between treatment groups at any time point (Fig 3C and 3D). There was a general decrease in total p38 protein over time in both treatment groups, however these differences were not statistically significant despite an approximately 50% reduction in total p38 from 1- to 12-weeks post-OVX. Further, E2 treatment significantly inhibited phospho-p38 levels, but only at the 1-week post-OVX time point (S2C and S2D Fig).

#### Brain–Ventral hippocampus

**Fig 4:**
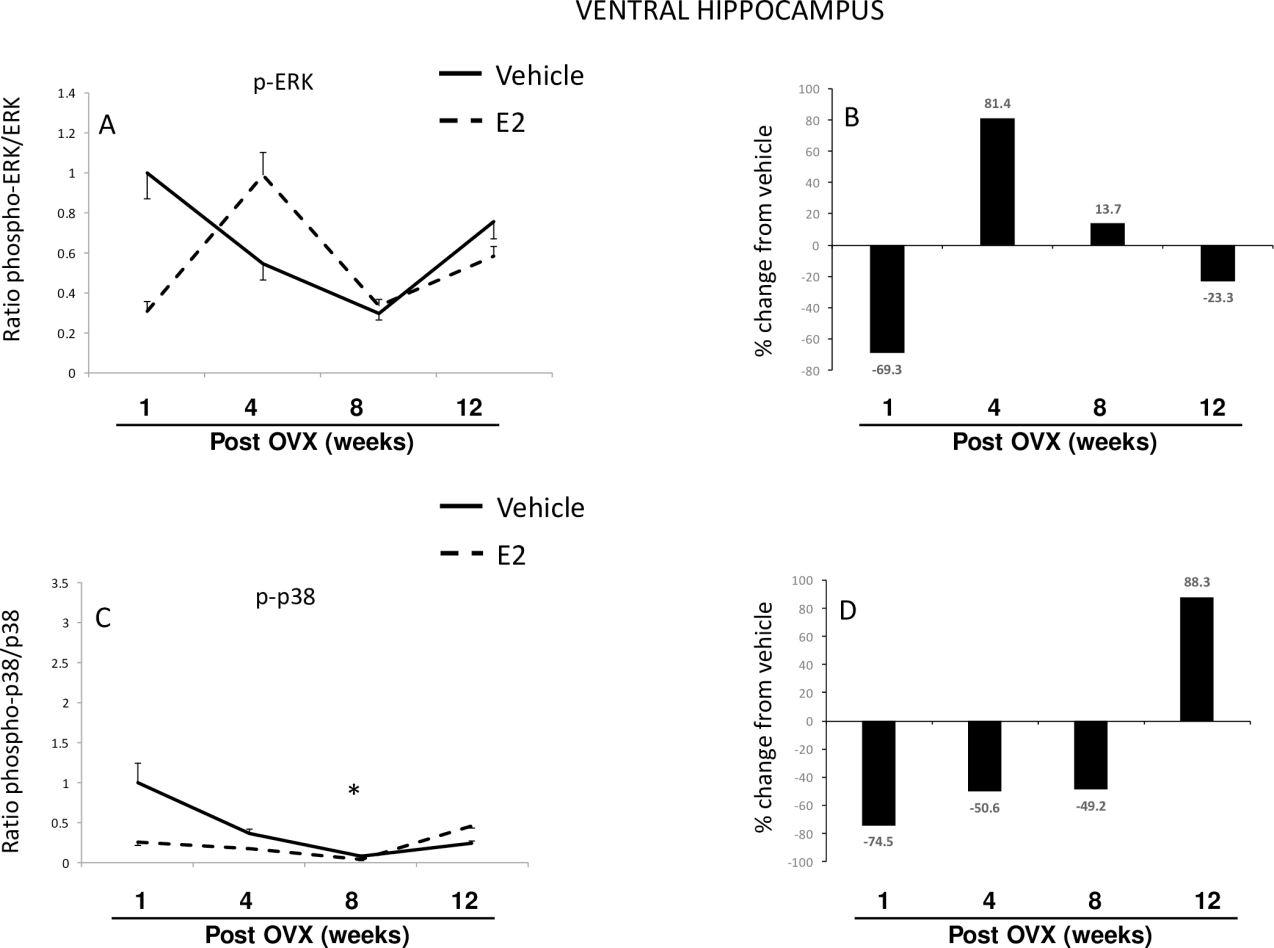
Effects of age and E2 treatment on ERK and p38 activation in the ventral hippocampus. Calculated ratio of phospho:total-ERK or p38 (A, C), and percent change from vehicle following E2 treatment (B, D). Data are expressed as mean fold change ± SEM compared to vehicle-treated animals at one week post-OVX (A, C). An * indicates statistically significant difference from 1-week time point; # indicates significant difference within the same time point.

A two-factor ANOVA revealed a significant interaction between length of E2 deprivation and subsequent E2 treatment on ERK, similar to the results from the other brain regions (hypothalamus and dorsal hippocampus; Table 1). Interestingly, the ratio of phospho:total ERK was not different between treatment groups, or with longer periods of E2 deprivation (Fig 4A), and this was likely due to parallel changes that occurred in total available ERK protein. Specifically, total ERK protein was significantly decreased at 4 weeks post-OVX in both vehicle and E2-treated animals, and these levels increased back to the levels observed at 1-week post-OVX by the 8 and 12-week time points (S3A Fig). The strong inhibitory action of E2 on ERK activation was only present at the early E2 deprivation time point (1 week post-OVX), while at later time points (4 and 8 weeks post-OVX) E2 treatment increased ERK activation (Fig 4B). Further, E2 treatment decreased phospho-ERK by 70%, at 1-week post-OVX, but both vehicle and E2-treated animals had significantly lower levels of phospoERK at the 4 and 8 weeks post-OVX time points (S3B Fig).

A two-factor ANOVA revealed a significant main effect of time on p38, but no significant interaction of time/treatment (Table 1). The ratio of phospho:total p38 was significantly decreased after 8 weeks of E2 deprivation, but there was no difference between treatment groups (Fig 4C). Overall, E2-treated animals had lower phosphorylated levels of p38 compared to vehicle treated animals until the 12-week post-OVX time point. At 12 weeks post-OVX there was a dramatic reversal with E2-treated animals having 88% higher levels of phosphorylated p38 compared to vehicle-treated animals (Fig 4D). E2 treatment significantly decreased the amount of total p38 protein at 12-weeks post-OVX, but had no effect on the amount of phosphorylated p38 (S3C and S3D Fig).

#### Heart–Left ventricle

**Fig 5:**
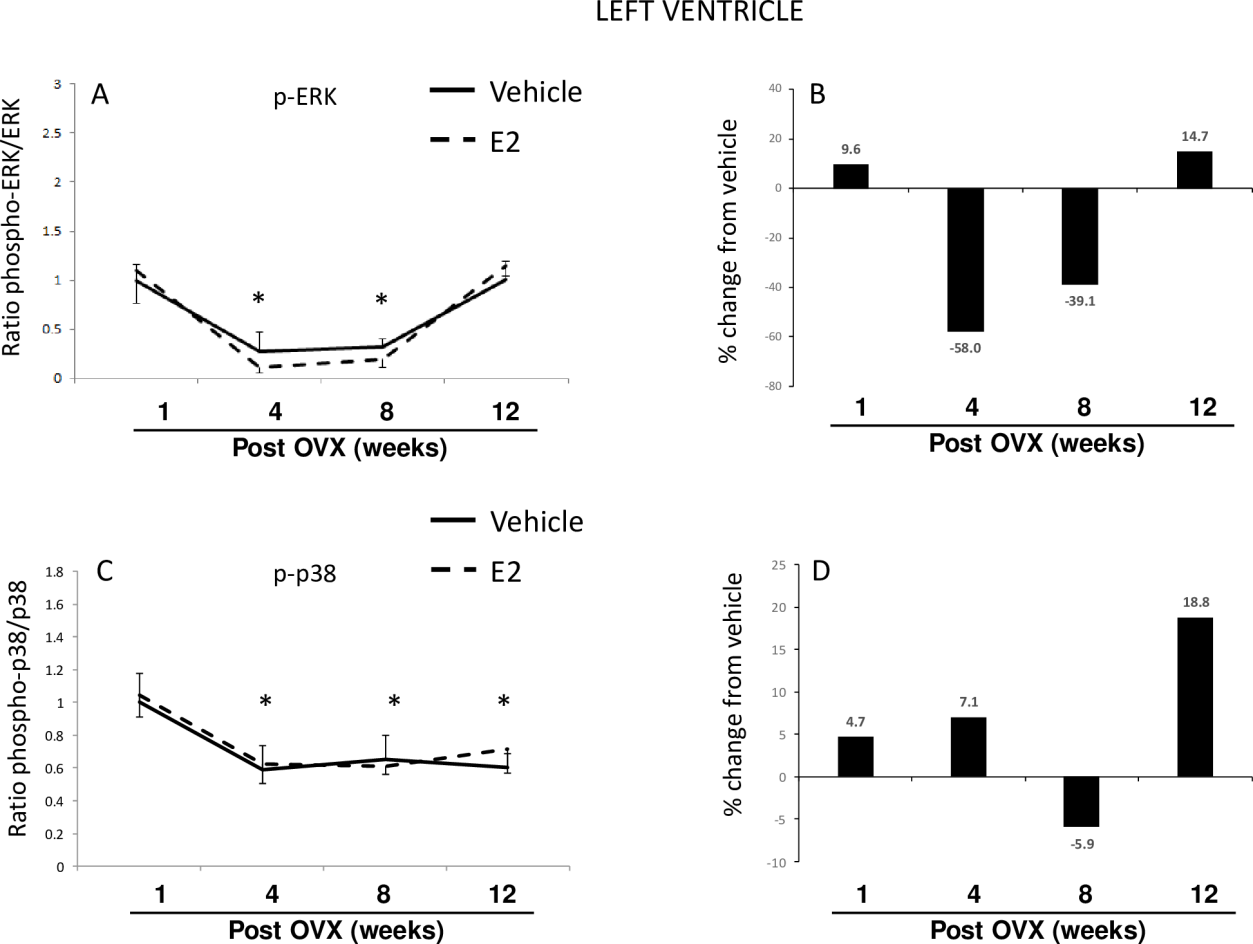
Effects of age and E2 treatment on ERK and p38 activation in the left ventricle. Calculated ratio of phospho:total-ERK or p38 (A, C), and percent change from vehicle following E2 treatment (B, D). Data are expressed as mean fold change ± SEM compared to vehicle-treated animals at one week post-OVX (A, C). An * indicates statistically significant difference from 1-week time point; # indicates significant difference within the same time point.

In contrast to results obtained in the brain, length of E2 deprivation (i.e. age alone) had the most striking effect on ERK activation, while E2 treatment had a modest, yet statistically significant effect. Moreover, a two-factor ANOVA revealed a significant interaction between length of E2 deprivation and subsequent E2 treatment (Table 1). After comparing the ratio of active to total ERK it was clear that the length of E2 deprivation was the most important factor regulating ERK activity (Fig 5A), as both treatment groups showed significant declines (80% decrease) at 4 and 8 weeks that returned to baseline by 12 weeks (Fig 5B). Notably, E2 treatment led to even further declines in active ERK (to nearly undetectable levels) at 4 and 8 weeks post-OVX (Fig 5B). Total ERK protein levels also increased at 12 weeks post OVX in the E2-treated animals compared to the vehicle-treated animals. In addition, phospho-ERK significantly decreased in both vehicle- and E2-treated groups at 4 and 8 weeks, yet there was a dramatic rebound in phospho-ERK in the E2-treated animals after 12 weeks of E2 deprivation (S4A and S4B Fig).

We next examined p38 and a two-factor ANOVA revealed there was a significant interaction between E2 treatment and length of time post-OVX on the levels of phospho-p38 in the heart (Table 1). There was also a strong main effect of time post-OVX, as the phospho:total p38 declined by more than 40% by the 4-week time point (Fig 5C). These lower levels were stable up to 12 weeks post-OVX, in E2 and vehicle treated animals, in contrast with ERK whose activation levels were restored 12 weeks post-OVX. Although E2-treatment significantly decreased phospho-p38 at 8 weeks post-OVX, this effect did not have a major impact on the amount of active p38 (Fig 5C and 5D). Total p38 protein levels were not significantly altered in the heart in our paradigm, however E2 treatment decreased phospho-p38 decreased at 8 weeks post-OVX (S4C and S4D Fig).

### Length of E2 deprivation and subsequent E2 treatment alters ERK and p38 mRNA expression

**Fig 6:**
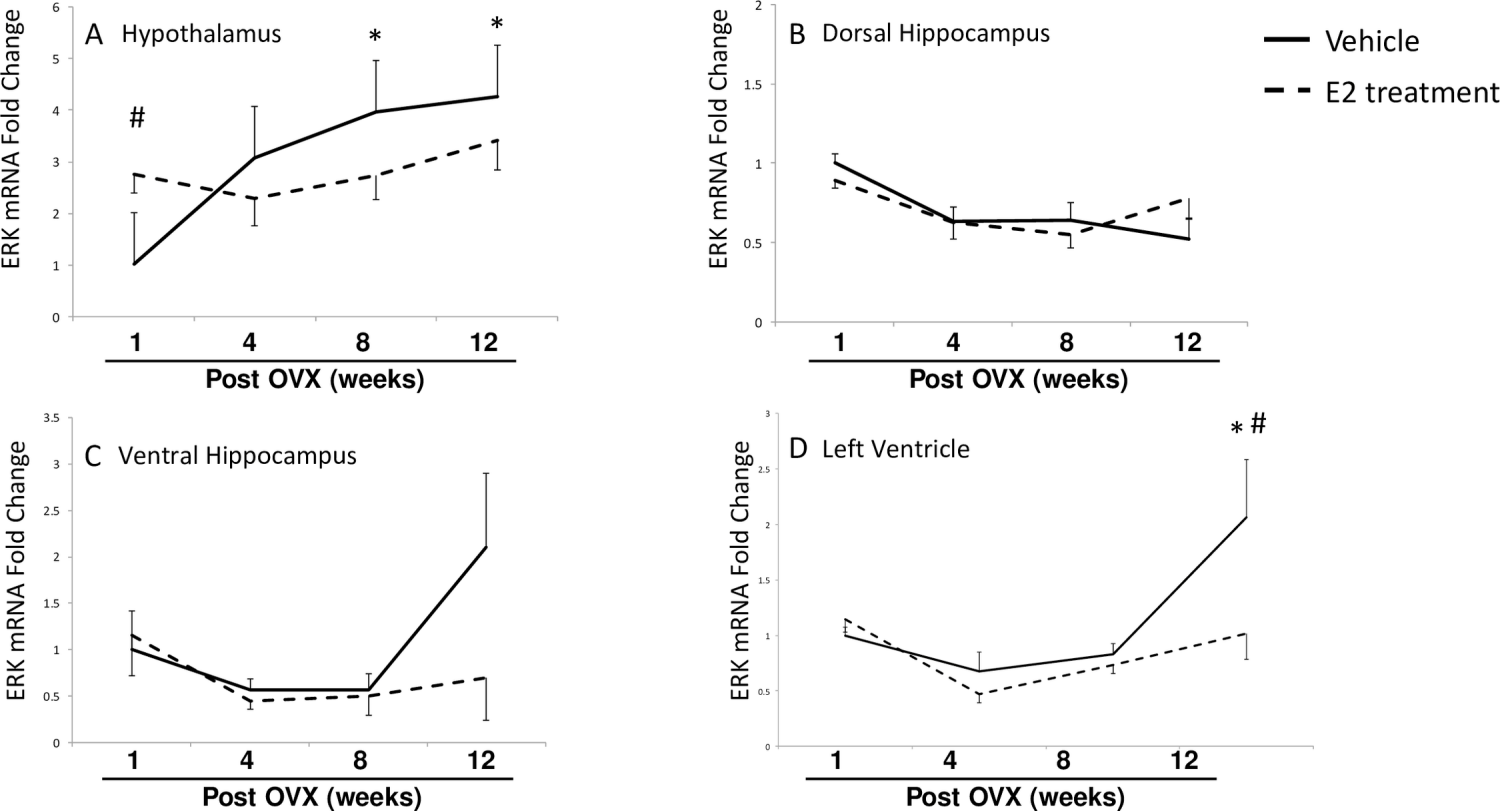
Effects of age and E2 treatment on ERK mRNA expression. ERK mRNA was measured using RT-qPCR. in the hypothalamus (A), dorsal hippocampus (B), ventral hippocampus (C), and left ventricle (D). Data are expressed as mean fold change ± SEM compared to vehicle-treated animals at one week post-OVX. An * indicates statistically significant difference from 1-week time point; # indicates significant difference within the same time point.

**Fig 7:**
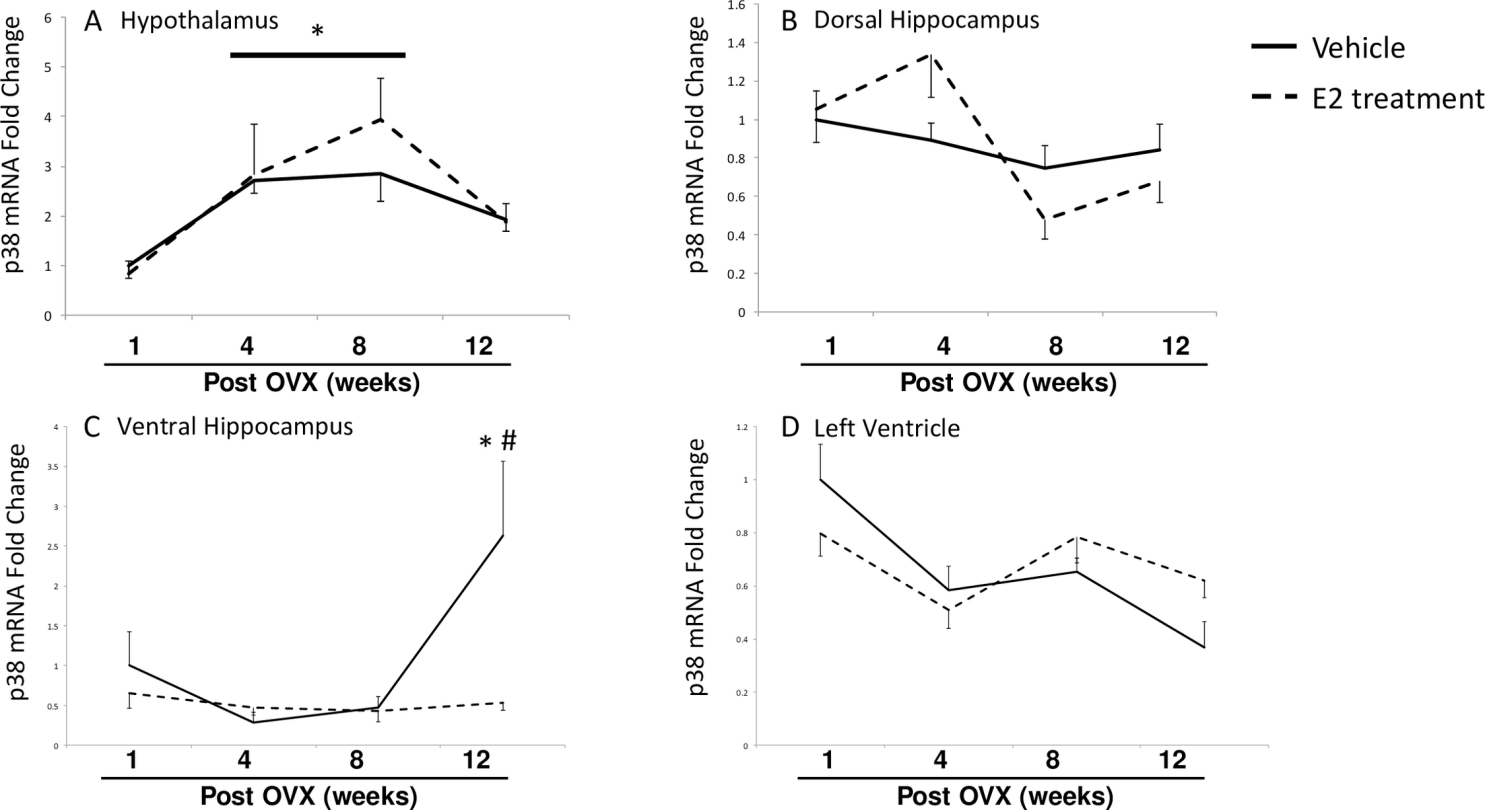
Effects of age and E2 treatment on p38 mRNA expression. P38 mRNA was measured using RT-qPCR. in the hypothalamus (A), dorsal hippocampus (B), ventral hippocampus (C), and left ventricle (D). Data are expressed as mean fold change ± SEM compared to vehicle-treated animals at one week post-OVX. An * indicates statistically significant difference from 1-week time point; # indicates significant difference within the same time point.

To determine if steady-state mRNA expression paralleled the observed changes in protein levels, we measured ERK (Fig 6) and p38 (Fig 7) mRNA in each brain region and in the left ventricle of animals subjected to our E2-deprivation paradigm. A two-factor ANOVA revealed statistically significant differences between treatment groups in the hypothalamus (Fig 6A) and the heart (Fig 6D), but not in either region of the hippocampus (Fig 6B and 6C). Specifically, ERK mRNA levels increased progressively over time in the hypothalamus, but this age-related increase was prevented by E2 treatment 8 and 12 weeks post OVX (Fig 6A). Conversely, in this same brain region E2 treatment increased ERK mRNA at the 1-week time point (Fig 6A, dashed line). In the left ventricle, ERK mRNA significantly increased after 12 weeks post-OVX, which was prevented by E2 treatment (Fig 6D).

Expression of p38 mRNA levels were also statistically different between groups subjected to our E2-deprivation paradigm (Fig 7). In the hypothalamus (Fig 7A) p38 expression was significantly increased after 4 and 8 weeks post-OVX, with no significant effect of E2 treatment. In the dorsal hippocampus a significant main effect of time, but not treatment was detected and pairwise comparisons did not reveal statistically significant changes between groups (Fig 7B). A significant interaction of treatment and time post-OVX was detected in the ventral hippocampus (Table 1), where E2 treatment significantly reduced p38 mRNA at the 12-week time point (Fig 7C). There were no differences between groups in p38 mRNA expression in the heart (Fig 7D).

## Discussion

MAPKs are central components of second messenger signaling pathways and are ubiquitously expressed in all cell types, therefore the functional implications for these findings are widespread. Here, we report the novel findings that E2 treatment differentially effects ERK and p38 activation, as well as total protein and mRNA expression, in the brain and the heart dependent on age and length of time following E2 deprivation (i.e. OVX/menopause). Importantly, length of E2 deprivation was a critical factor in every parameter analyzed. For example, ERK and p38 were significantly less active 4 or more weeks following OVX regardless of treatment in the heart, possibly reflecting an age-related change. Age has been shown to both increase and decrease MAPKs activation, depending on tissue analyzed, sex, age and species of the animal model, as well as experimental design [26, 56–58]. For instance, Li et al. hypothesized that p38, a key regulator of pro-inflammatory cytokine biosynthesis, would be activated by the low grade inflammation that is associated with the aging process. They observed increased levels of inflammatory markers accompanied by doubling in p38 activation in the lung and whole brain homogenate of old (20 mo.) compared to young (2 mo.) C57BL/6J mice [58]. Similarly, p38 expression and activity was increased by 2.5 fold in the brain of 26 mo. compared to 2 mo. Fisher 344 rats [26]. These findings align with our results in the hypothalamus where p38 had a 2.5 fold increase 12 weeks post-OVX, regardless of treatment. However, these results were brain region specific, as we did not observe age-related changes for phospho-p38 in other brain regions analyzed.

Perhaps most intriguing was the observation that E2-induced changes in activated ERK and p38 were prolonged, given that the last dose of E2 was administered 24 hours before euthanasia. The prevailing view is that the non-genomic actions of E2 occur within minutes and the effects on MAPK phosphorylation are transitory. Indeed, this has been observed repeatedly *in vitro*, however accumulating evidence suggests that the *in vivo* regulation of MAPKs is more complex [24, 29, 59–65]. One possibility is that E2, acting through classical genomic pathways, altered cellular components required for maintaining balance between active and inactive MAPKs. For example, crosstalk between phosphorylation and ubiquitination pathways can exert long-term changes in cellular processes through multiple feedback loops that ultimately impact apoptosis and cell proliferation [66]. Moreover, we and others have shown that E2 can regulate microRNAs in the brain and the heart, and some of these miRNAs could target components of MAPK signaling pathways providing a putative mechanism for sustained activation of these kinases [50, 67–70]. These results were further corroborated in a 2013 study which showed E2 could regulate long-term activation of MAPKs by altering expression of microRNAs that silence upstream inhibitors of ERK activation [70]. The findings herein combined with other published studies suggest that E2 can exert prolonged changes in MAPKs activation that cannot be explained by the acute non-genomic actions of E2 alone.

The significant interactions observed between the two factors of time and E2 treatment for ERK, but not p38, in nearly every brain region and in the heart could have important functional ramifications for understanding the physiological consequences of ET in postmenopausal women. Specifically, the timing of E2 treatment would not be expected to impact p38 signaling in these tissues, despite the fact p38 activity was independently altered by E2 and age. Conversely, activated ERK was mainly decreased following E2 treatment and the timing of E2 treatment dictated the magnitude of decline. Activation of ERK induces downstream signaling pathways that mediate both neuro- and cardioprotection. For instance, the formation of dendritic spines was increased by E2 treatment through ERK-mediated mTOR (mammalian target of rapamycin) and this E2-induced synaptic plasticity is a key mechanism underlying memory consolidation and storage [71]. Similarly, activated ERK mediates cardioprotective pathways in the heart and phospho-ERK is increased by E2 in young adult rat cardiomyocytes [25, 29, 72–74]. The E2-induced decrease of activated ERK in the brain and heart following longer periods post-OVX reveal a putative mechanism for memory decline and reduced cardioprotection following ET in late postmenopausal women.

Increased estrogen receptor-mediated gene transcription is the most likely explanation for our observed increases in total ERK and p38 protein levels. Indeed, we found that the mRNA levels of both kinases were significantly altered by E2 treatment and length of deprivation in the brain and heart. Sequence analysis of the ERK and p38 gene promoters revealed an abundance of binding sites for several transcription factors such as NF-kB and GATA families as well as multiple estrogen response elements (ERE) [75]. The canonical actions of ERs are through direct ERE binding, however ERs can also regulate transcription by tethering to other transcription factors, such as members of the Jun and Fos families acting at AP-1 sites (activator protein-1) [76]. Notably, there are 28 and 34 identified AP-1 sites within 2000 bp upstream of the p38 and ERK translation start sites, respectively. These data demonstrate that there are multiple mechanisms for E2 to regulate ERK and p38 transcription directly at the level of the gene promoter.

In the last decade, sophisticated genomic and proteomic tools have allowed for the quantitative molecular analysis of complex biological samples. These experiments revealed low correlations between mRNA and protein levels in many samples [77]. Similarly, changes in ERK and p38 protein levels did not tightly correlate with their altered mRNA levels in our paradigm. This suggests that age and/or E2 treatment can regulate compensatory factors that affect protein translation or stability. For example, we observed a 3 to 5- fold increase of ERK mRNA following prolonged E2 deprivation in the hypothalamus, yet total ERK protein was unchanged. This could be partly explained by the semi-quantitative nature of the techniques (i.e. Western blot). Alternatively, this observation could be due to increased mRNA turnover or translational inhibition. Several mechanisms of post transcriptional regulation of mRNA have been described and microRNAs are interesting example of regulatory molecules that can repress target mRNA translation [78]. Our recent work demonstrated that microRNA expression is differentially regulated by prolonged E2 deprivation and subsequent E2 treatment using this same animal treatment paradigm in aged rats [50, 68]. In line with the current findings, several of those E2-regulated microRNAs have the potential to inhibit p38 and ERK mRNA translation, resulting in decreased protein levels. We also identified a subset of microRNAs that are differentially regulated by E2 in young (3 mo.) vs. old (18 mo.) rats using a microRNA microarray platform [68]. Bioinformatics pathway analyses revealed that the MAPK pathway was predicted as the most represented cellular pathway targeted by the microRNAs we identified as E2 regulated [68]. Regulation of microRNAs is just one possible explanation for the discrepancies seen between mRNA and protein levels in complex biological samples.

Our paradigm cannot rule out the possibility that brain-derived estrogens affected phosphorylation and/or total ERK p38 protein levels. The brain is an extra gonadal site of steroid hormone synthesis and it has been demonstrated that E2 is synthesized *de novo* from cholesterol in certain brain regions [79]. The levels of estrogens in the brain are lower in post-menopausal women compared to pre-menopausal women, but do not further decrease with advanced age [80]. Our study did not measure the concentration of total estrogens in the brain or cerebrospinal fluid and therefore, cannot rule out possible contributing effects from this extra gonadal source. However, it is expected that the rate of synthesis and quantities of estrogens produced in the brain would not differ between the vehicle and E2 treated groups. Measuring estrogens in the brain or cerebrospinal fluid poses technical challenges that were beyond the scope of the current study.

Despite the understanding of the importance of the time of initiation of ET in women for neuroprotection and cardioprotection gained from clinical studies, clear mechanistic insight is still lacking. We designed our study to model a main tenet of the timing hypothesis, as based on clinical observations [11, 81, 82]. One limitation is that rodent reproductive senescence is not comparable to the menopausal transition in women [83, 84]. However, the surgically-induced menopause model used in rodents is the most accurate method to determine the length of time following total ovarian hormone depletion, and surgically-induced menopause is clinically relevant for some women. We have also demonstrated previously that 18 months of age is a physiologically relevant comparison to human in this strain of rat (Fisher 344), although Sprague Dawley rats demonstrate much earlier reproductive senescence [47]. The concepts referred as “window of opportunity” or “timing hypothesis” emerged from clinical studies that found that age of ET initiation determined the successful outcome of the study. Indeed estrogens reduced risk of cognitive decline and dementia when administered to women in early stage of menopause [85]. Postmenopausal women receiving ET in a 2012 Danish study also had significantly reduced risk of mortality and heart failure, without an increased risk of breast cancer or stroke [86]. The WHI ET follow-up showed that women 50–59 of age had statistically significant reduction in coronary heart disease (Hazard Ratio HR of 0.59), myocardial infarction (HR 0.54) and overall mortality (0.73) [87]. These are only a few examples of how cognitive and cardiovascular health of menopausal women can be improved if ET is started at the right time (i.e. in early menopause). Our data highlight MAPKs as a possible focus of further analysis, as these kinases are critical regulators of cell signaling pathways.

## Supporting Information

S1 Fig Effects of age and E2 treatment on ERK and p38 protein expression and activation in the hypothalamus. Representative blots and fold change of total ERK protein (A), phosphorylated ERK (B), total p38 (C) and phosphorylated p38 (D). Data are expressed as mean fold change ± SEM compared to vehicle-treated animals at one week post-OVX. An * indicates statistically significant difference from 1-week time point; # indicates significant difference within the same time point.
(PPTX)

S2 Fig Effects of age and E2 treatment on ERK and p38 protein expression and activation in the dorsal hippocampus. Representative blots and fold change of total ERK protein (A), phosphorylated ERK (B), total p38 (C) and phosphorylated p38 (D). Data are expressed as mean fold change ± SEM compared to vehicle-treated animals at one week post-OVX. An * indicates statistically significant difference from 1-week time point; # indicates significant difference within the same time point.
(PPTX)

S3 Fig Effects of age and E2 treatment on ERK and p38 protein expression and activation in the ventral hippocampus. Representative blots and fold change of total ERK protein (A), phosphorylated ERK (B), total p38 (C) and phosphorylated p38 (D). Data are expressed as mean fold change ± SEM compared to vehicle-treated animals at one week post-OVX. An * indicates statistically significant difference from 1-week time point; # indicates significant difference within the same time point.
(PPTX)

S4 Fig Effects of age and E2 treatment on ERK and p38 protein expression and activation in the left ventricle. Representative blots and fold change of total ERK protein (A), phosphorylated ERK (B), total p38 (C) and phosphorylated p38 (D). Data are expressed as mean fold change ± SEM compared to vehicle-treated animals at one week post-OVX. An * indicates statistically significant difference from 1-week time point; # indicates significant difference within the same time point.
(PPTX)
