# Supplementary material for: Differential Effects of E2 on MAPK Activity in the Brain and Heart of Aged Female Rats
Source: PLoS One. 2016 Aug 3;11(8):e0160276. doi: 10.1371/journal.pone.0160276 (PMC4972350; doi:10.1371/journal.pone.0160276)
Supplement: S4 Fig — Representative blots and fold change of total ERK protein (A), phosphorylated ERK (B), total p38 (C) and phosphorylated p38 (D). Data are expressed as mean fold change ± SEM compared to vehicle-treated animals at one week post-OVX. An * indicates statistically significant difference from 1-week time point; # indicates significant difference within the same time point. (PPTX) [file pone.0160276.s004.pptx]

## Slide 1
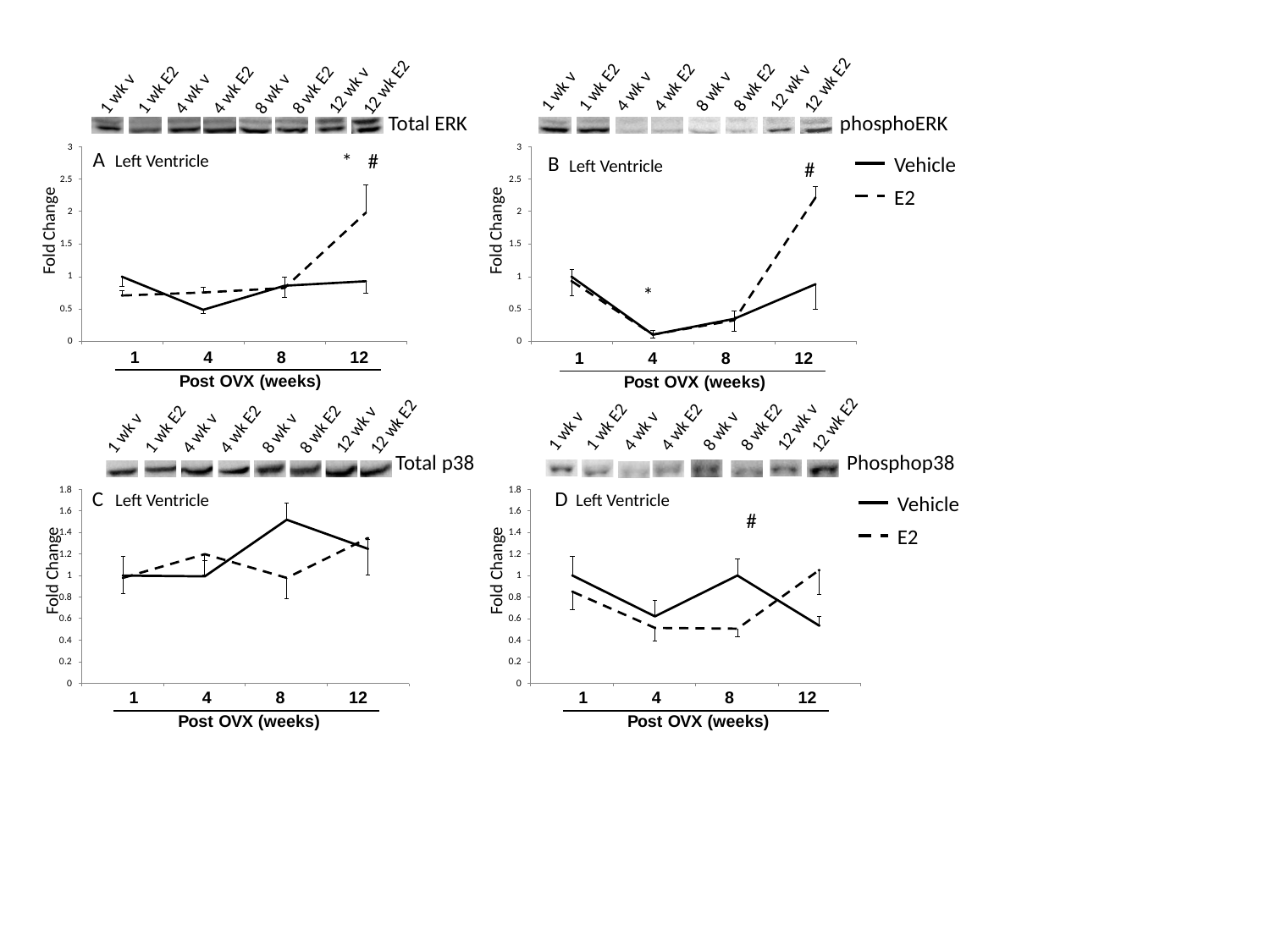

12 wk E2
12 wk v
1 wk v
1 wk E2
4 wk v
4 wk E2
8 wk v
8 wk E2
phosphoERK
12 wk E2
12 wk v
1 wk v
1 wk E2
4 wk v
4 wk E2
8 wk v
8 wk E2
Total ERK
A
Left Ventricle
B
*
#
Left Ventricle
#
Vehicle
E2
Fold Change
Fold Change
*
12 wk E2
12 wk v
1 wk v
1 wk E2
4 wk v
4 wk E2
8 wk v
8 wk E2
Phosphop38
12 wk E2
12 wk v
1 wk v
1 wk E2
4 wk v
4 wk E2
8 wk v
8 wk E2
Total p38
C
D
Left Ventricle
Left Ventricle
Vehicle
E2
#
Fold Change
Fold Change
